# Squeeze Film Air Damping in Tapping Mode Atomic Force Microscopy

**DOI:** 10.3390/mi8070226

**Published:** 2017-07-20

**Authors:** Yang Zhao, Qiangxian Huang, Liansheng Zhang, Yong Zhang, Rongjun Cheng

**Affiliations:** 1 School of Instrument Science and Opto-electronics Engineering, Hefei University of Technology, Hefei 230009, China; zhaoyang@ahjzu.edu.cn (Y.Z.); lszhang@hfut.edu.cn (L.Z.); zhangy@hfut.edu.cn (Y.Z.); chengrj@hfut.edu.cn (R.C.); 2 School of Electronics and Information Engineering, Anhui Jianzhu University, Hefei 230601, China

**Keywords:** squeeze film air damping, damping coefficient, atomic force microscopy, dynamic plowing lithography

## Abstract

In dynamic plowing lithography, the sample surface is indented using a vibrating tip in tapping mode atomic force microscopy. During writing, the gap between the cantilever and the sample surface is very small, usually on the order of micrometers. High vibration frequency and small distance induce squeeze film air damping from the air in the gap. This damping can cause variations in the cantilever’s vibrating parameters and affect the accuracy of the nanoscale patterning depth. In this paper, squeeze film air damping was modeled and analyzed considering the inclined angle between the cantilever and the sample surface, and its effects on the resonant amplitude and damping coefficient of the cantilever were discussed. The squeeze film air damping in the approaching curve of cantilever was observed, and its effect on fabricating nanopatterns was discussed.

## 1. Introduction

Tapping mode atomic force microscopy (AFM)-based nanolithography has been widely used for structuring materials and fabricating nanopatterns [1,2]. Dynamic plowing lithography (DPL) is one of the robust methods [3,4,5]. In the DPL process, the cantilever—which is driven by a dither piezo actuator—vibrates near its resonance frequency. The image of the sample topography can be obtained by recording and regulating the modulation amplitude applied to the dither piezo that drives the cantilever oscillations. The sample can be written by changing the modulation amplitude [6]. The precise control of vibration amplitude is a key factor in keeping the accuracy of patterning depth [7]. The distance between the cantilever and the sample surface during the nanolithography process is very small, and usually on the order of micrometers [8]. Cantilever vibration makes air flow in and out of the gap between the cantilever and the sample surface, which leads to squeeze film air damping. This damping can cause variations in the cantilever’s vibrating parameters, such as vibrating amplitude and quality factor, and errors in nanometer scale patterning.

Hoummady [9] observed the air damping effect in experiments, and found that the amplitude of the cantilever varies slightly when the sample is and is not placed under the cantilever. However, the phenomenon was simply discussed but not analyzed systematically. Bowen [10] studied the force applied to a cantilever due to fluid squeezing in contact mode. Gunther [11] studied air damping in tuning fork vibrations. In Gunther’s model, the tip and the sample were simplified into a sphere and a plate. Damping was analyzed, but the damping between the tuning fork cantilever and the sample was not discussed. Combining the Lorentz equation and the theory of mechanics, Leveque [12] studied the effect of air damping on cantilever amplitude when the cantilever operates at low frequency. However, the effects of air damping on the damping coefficient and the quality factor were not reported. Pandey [13] presented an analytical model that provides the squeeze film damping values for the cantilever resonators of micro-electro-mechanical systems (MEMS). The effects of squeeze damping on cantilever amplitude and damping coefficient were discussed.

In these studies, the cantilevers and samples were simplified into parallel plates. However, an angle of 12.5° usually existed between them in AFM, which made the models’ illustration of the actual damping ratio inaccurate. When the cantilever approaches the sample, the small change in damping ratio affects the characteristic parameter of the cantilever vibration and the measurement characteristics. Therefore, the air damping between the cantilever and the sample that resembles actual conditions should be modeled and analyzed. In this study, a slant cantilever model was established, the squeeze film damping between the cantilever and the sample was deduced, and the main factor of damping ratio and cantilever amplitude was analyzed. The model followed practical situations because the angle of inclination was considered. Corresponding experiments were performed in tapping mode AFM, and theoretical analysis results were verified.

## 2. Squeeze Film Damping of the Cantilever

### 2.1. Model for Squeeze Film Damping

A rectangle cantilever is usually regarded as a linear elastic beam with uniform sections. One end of the cantilever is fixed, while the other is driven to oscillate near its free resonant frequency through excitation, such as static electricity and machinery excitation.

**Figure 1:**
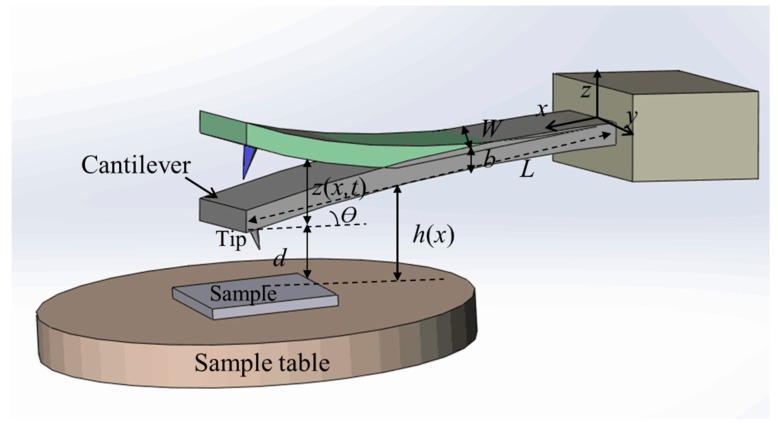
Schematic of simplified cantilever and sample model.

When the tip of the cantilever taps the sample surface, the distance between them is small. During oscillation, the gas film between them flows in and out repeatedly and generates the squeeze film effect, which induces extra damping and stiffness. The common dimensions of a cantilever are as follows: length is several hundreds of micrometers, width is dozens of micrometers, and thickness is several micrometers. The amplitude is several nanometers to approximately 1 µm. The tip length is about ten micrometers, and its radius is smaller than ten nanometers. Compared with the cantilever, the tip is very small and can be ignored. Then the oscillating rectangle cantilever can be simplified to a rectangular plate. The cantilever and sample model was simplified to two plates as shown in Figure 1.

In Figure 1, *W*, *b*, and *L* are the width, thickness, and length, respectively. *z*(*x*, *t*) is the cantilever deflection. When inertia and lateral displacement are not considered, the squeeze film damping effect of the cantilever and sample can be expressed by the Reynolds equation as follows:

(1) $$\frac{\partial }{\partial x}\left(\rho \frac{h^{3}}{\mu }\frac{\partial p}{\partial x}\right)+\frac{\partial }{\partial y}\left(\rho \frac{h^{3}}{\mu }\frac{\partial p}{\partial y}\right)=12\left(h\frac{\partial \left(p\right)}{\partial t}+p\frac{\partial \left(h\right)}{\partial t}\right)$$

where *p* is the pressure in the film, *ρ* is the density of air, *µ* is the coefficient of the fluid’s viscosity, and *h* is the thickness of the film. Gas density is directly proportional to its pressure under isothermal conditions. The gas film in tapping mode AFM was considered incompressible, and the change of pressure with time is zero. Then, Equation (1) can be transformed to

(2) $$\frac{\partial }{\partial x}\left(\frac{\rho h^{3}}{\mu }\frac{\partial p}{\partial x}\right)+\frac{\partial }{\partial y}\left(\frac{\rho h^{3}}{\mu }\frac{\partial p}{\partial y}\right)=12\frac{\partial \left(hp\right)}{\partial t}$$

where *p* consists of two components (*p* = *P_a_* + Δ*p*): *P_a_* is the ambient pressure, and Δ*p* is the deviatory pressure caused by the squeeze film effect. Film thickness can be expressed as

(3) h=d+(L−x)⋅sinθ+z(x,t)

where *d* and *θ* are the distance and the angle between the cantilever and the sample surface, respectively. If D=d+(L−x)⋅sinθ, then *h* can be expressed as *h* = *D* + *z*(*x*, *t*).

The squeeze film effect consists of two parts: viscous and elastic damping forces [14]. If the plate oscillates with a low frequency, the gas film is not compressed and escapes. In this case, viscous damping force is dominant. If the plate oscillates at high frequency, the gas film is compressed and contained. In this case, elastic damping force is dominant. At the cut-off frequency, the viscous damping force is equal to the elastic damping force. The cut-off frequency is given as [15]:

(4) $$\omega_{c}=\frac{\pi^{2}h_{0}^{2}P_{a}}{12\mu W^{2}}$$

In Equation (4), the atmosphere is *P_a_* = 1.013 × 10^5^ Pa, and the effective dynamic viscosity is *μ* = 1.81 × 10^−5^ Pa·s. In the experiment, the width *W* was several dozens of micrometers, the initial thickness of the gas film *h*_0_ was several micrometers, and the calculated cut-off frequency was several MHz. Since the resonance frequency of the cantilever was not more than several hundred kHz, it was much lower than the cut-off frequency. Therefore, the gas film in tapping mode AFM was considered incompressible, and the viscous damping force was dominant.

If the cantilever oscillates with a small amplitude around its balance position, then Δ*h* << *h*_0_, Δ*p* << *P_a_*. The air between the two plates should be considered incompressible, and Equation (3) should be substituted into Equation (2), then Equation (2) can be transformed to

(5) $${\left(D^{\prime }\right)}^{3}\frac{\partial^{2}\left(\Delta p\right)}{\partial x^{2}}+{\left(D^{\prime }\right)}^{3}\frac{\partial^{2}\left(\Delta p\right)}{\partial y^{2}}+\left[-3{\left(\sin\theta \right)}^{3}\cdot x^{2}+6D^{\prime }\cdot x\cdot \sin\theta +3{\left(D^{\prime }\right)}^{2}\right]\left(\frac{\partial \left(\Delta p\right)}{\partial x}\right)=12\mu \left(\frac{\partial z}{\partial t}\right)$$

where *x* is the displacement in the *x*-direction, and $D^{\prime }=d+L\cdot \sin\theta$. The cantilever oscillates by rolling in the *y* axis. Thus, the pressure in the y direction shows a small or no change and $\frac{\partial \left(\Delta p\right)}{\partial y}=0$. Both *θ* and sin*θ* are small. Hence, Equation (5) can be simplified as

(6) $${\left(D^{\prime }\right)}^{3}\frac{\partial^{2}\left(\Delta p\right)}{\partial x^{2}}+\left[3{\left(D^{\prime }\right)}^{2}\right]\left(\frac{\partial \left(\Delta p\right)}{\partial x}\right)=12\mu \left(\frac{\partial z}{\partial t}\right)$$

By combining the boundary conditions of the cantilever (*x* = 0, Δ*p* = 0; *x* = *L*, Δ*p* = 0), we can obtain

(7) $$\Delta p=\left[-\frac{e^{-\frac{3}{D^{\prime }}x}}{e^{-\frac{3}{D^{\prime }}L}-1}+\frac{x}{L}+\left(e^{-\frac{3}{D^{\prime }}L}-1\right)\right]\cdot \frac{4\mu L}{{\left(D^{\prime }\right)}^{2}}\left(\frac{\partial z}{\partial t}\right)$$

Damping coefficient *c* can be calculated as

(8) $$c=\left(\frac{3L}{2}+\frac{D^{\prime }}{3}\right)\cdot \frac{4W\mu L}{{\left(D^{\prime }\right)}^{2}}$$

The above equations show that the main factors of the squeeze film damping coefficient are the physical cantilever dimensions, the distance between the cantilever and the sample, and the atmospheric viscosity coefficient. The squeeze film damping coefficient is approximately proportional to the length and the width of the cantilever. For specific cantilevers in specific environments, the physical dimension, temperature, humidity, and pressure are constant. Therefore, cantilever–sample distance is the critical factor, and the squeeze film damping coefficient is inversely proportional to the cantilever–sample distance.

### 2.2. Factor of the Cantilever Squeeze Film Damping

**Figure 2:**
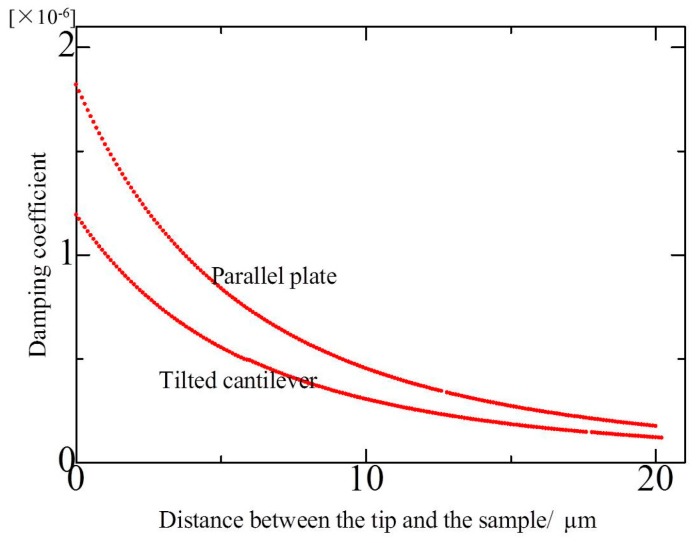
Dependence of damping coefficient on the tip–sample distance.

To observe the effect of cantilever–sample distance on squeeze film damping, the simulation curve of Equation (8) was plotted. The experimental parameters were as follows: cantilever width was *W* = 28 μm, cantilever length was *L* = 225 μm, atmospheric viscosity coefficient was *μ* = 1.81 × 10^−5^ Pa·s. The tip length was 17 μm, so the smallest distance of the cantilever and the sample was 17 μm. Then, in the simulation, the range of the tip–sample distance was 0–20 μm; that is, the cantilever–sample distance was 17–37 μm. For comparison with the tilted cantilever model, the damping coefficient in parallel plate was calculated according to Reference [15]. The results are shown in Figure 2.

As shown in Figure 2, tip–sample distance mainly influences the squeeze film damping coefficient. With the distance decreasing, the squeeze film damping occurs and increases. When the distance is larger than 15 μm, the damping coefficient keeps approximately constant. When the distance is between 10–15 μm, the squeeze film damping ratio increases slowly, which indicates that squeeze film damping occurs between the cantilever and the sample. When the distance is smaller than 10 μm, the squeeze film damping ratio increases rapidly, which indicates the significant increase of squeeze film damping in the cantilever and the sample.

It can be seen that the general change trends were similar in the two models. However, the values of damping coefficient are smaller and change less sharply than that of the parallel plate.

### 2.3. Dynamic Amplification Factor of the Cantilever

The cantilever vibration system is a single-degree system, and the dynamic amplification factor can be expressed as

(9) $$\mu_{Am}=\frac{1}{\sqrt{{\left(1-\frac{\phi^{2}}{\omega^{2}}\right)}^{2}+{\left(2c_{sum}\frac{\phi }{\omega }\right)}^{2}}}$$

where *μ_Am_* is the dynamic amplification factor, which is the ratio of the maximum dynamic displacement to the static displacement [16], *c_sum_* is the total damping coefficient, *ω* is the resonant frequency, and *φ* is the frequency of the external excitation force. The relationship of *ω* and *φ* is usually expressed as *φ* = 0.998·*ω*. Thus,

(10) $$\mu_{Am}\approx \frac{1}{2c_{sum}}$$

In Equation (10), the cantilever amplitude is inversely proportional to the damping coefficient. Cantilever dissipation during vibration can be divided into two parts [17]. The first part is caused by lattice and surface imperfections and phonon-phonon scattering, which is expressed by *c_int_*. The second part is caused by the interaction between the cantilever and the external ambient, which is denoted by *c*. The total damping coefficient can be calculated by *c_sum_* = *c_int_* + *c*. The damping coefficient caused by internal energy dissipation can be considered a fixed value because the frequency and amplitude variations during cantilever vibration are minimal [18]. As the cantilever–sample distance decreases, the squeeze film damping coefficient increases. This phenomenon leads to the increase in the general damping coefficient of the cantilever and the decrease in the dynamic magnification coefficient and amplitude of the cantilever. In microfabrication, the cantilever approaches the sample, the variation in the oscillation amplitude induces the fabricating depth error in nanopatterning.

## 3. Experiment Results and Discussion

### 3.1. Experimental System Construction

**Figure 3:**
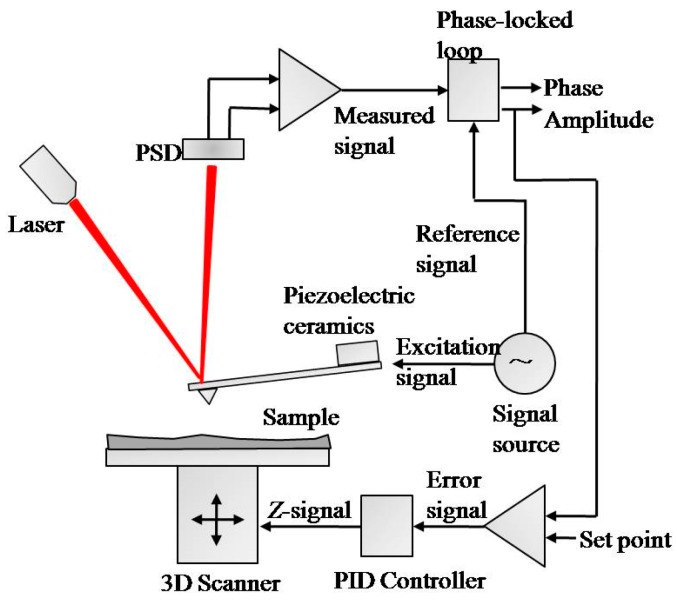
Structure of the experimental system. (PSD: Position Sensitive Detector; PID controller: Proportion Integration Differentiation controller.)

The schematic of the experimental system is shown in Figure 3. The cantilever is oscillated near its resonant frequency using piezoelectric ceramics, thereby causing its tip to vibrate at constant amplitude along the *z* axis. The amplitude is tested by the optical lever and processed by a signal processing system to obtain the corresponding voltage signal.

**Figure 4:**
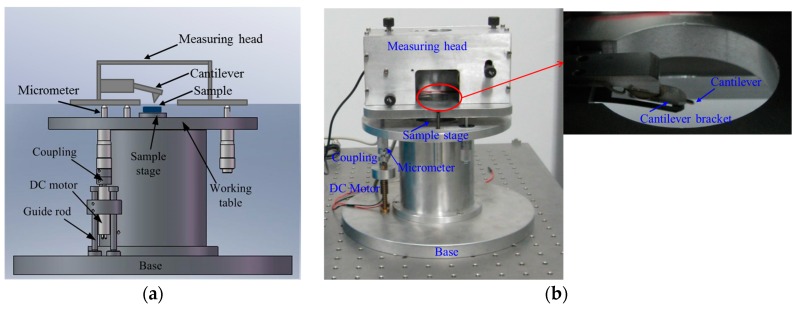
The cantilever–sample approach system: (**a**) Schematic diagram (**b**) Experimental set-up.

The tip–sample distance is adjusted by the cantilever–sample approach system, as shown in Figure 4. The system consists of a piezoelectric tube, a direct current (DC) motor, a reduction gear box, and a micrometer screw. The cantilever is fixed in the measuring head which is placed on the micrometer. The approach process is divided into two parts: fine approach (tip–sample distance is within 0–2 μm) and quick approach (tip–sample distance is more than 2 μm).The fine approach is performed by the piezoelectric tube, while the quick approach is performed by the motor, the reduction gear box, and the micrometer screw. The cantilever–sample distance is changed by controlling the measuring head in quick approach or the sample stage in fine approach to move up and down.

In the fine approach, the piezoelectric tube is enveloped in the sample stage and its maximum extension is 2 μm. When the length of the piezoelectric tube changes, the sample placed on the stage is moved up and down correspondingly. The extension of the piezoelectric tube is determined by the voltage applied on it, and the tip–sample distance is adjusted in the range of 0–2 μm.

In the quick approach, the motor is connected to the micrometer by coupling, which drives the micrometer to move up and down along the guide rod direction to ensure that the tip–sample distance changes correspondingly. A high-precision micrometer and a reduction gear box are used in this approach to adjust the distance and ensure the stability. To perform the quick approach, the maximum voltage is firstly applied on the piezoelectric tube to obtain the maximum extension (2 μm). Then, the motor is kept running until the cantilever’s tip taps the sample and the distance becomes 0 μm. Then, the motor is stopped, the voltage is removed, and the original length of the piezoelectric ceramic tube is restored. At this point the tip–sample distance is 2 μm. From the 2 μm position, the motor is controlled to move the cantilever away from the sample, thereby increasing the distance. Distance is determined by the motion time, the parameters of the motor, and the reduction gear box.

### 3.2. Test of the Effect of Squeeze Film Damping in Tapping Mode AFM

**Figure 5:**
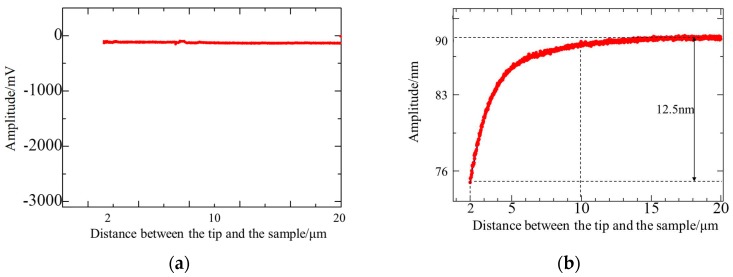
Approach curve of the 0.2 N/m cantilever (**a**) in contact mode (**b**) in tapping mode.

Experiments were designed to test the effect of squeeze film damping. A ContAl (Budget Sensors, Sofia, Bulgaria) cantilever was used in the experiments. Its nominal elastic constant and geometric sizes were 0.2 N/m and 450 μm × 50 μm × 2 μm (length, width, and thickness). This cantilever is sensitive to small external forces and the squeeze film damping effect. To compare the squeeze film damping effects in contact and tapping modes, the cantilever was driven to work in contact and tapping mode in the same environment. The cantilever was controlled by the motor to approach the sample gradually and the tip–sample distance was in the range of 2–20 μm. Within this scope, the amplitude of the cantilever was tested and the force–displacement curve in the approach was recorded to observe the force of the cantilever. Test curves in contact and tapping modes are shown in Figure 5a,b respectively.

In Figure 5a, the cantilever is static in contact mode. Cantilever amplitude was practically unchanged in the approach of the cantilever–sample, which validated that the cantilever was not affected by any external force or Van der Waal’s force.

In Figure 5b, the cantilever is in tapping mode. The cantilever resonated with a frequency of 15.4 kHz, and the free oscillation amplitude was about 90 nm. The deflection sensitivity was 14.5 V/μm. When the tip–sample distance reached higher than 15 μm, the amplitude was kept constant. When the distance reached higher than 10 μm, the cantilever amplitude decreased slightly as the distance decreased. When the distance was between 2–10 μm, the cantilever amplitude decreased significantly because of external force generation during the approach. Reduction was approximately 12.5 nm, which was about 14% of the free amplitude. The reaction range [19] of Van der Waal’s force was within several to hundreds of nanometers, thus indicating that the distance in the experiment was out of region. At the same time, the test result at contact mode showed that external forces were not present during the approaching process. Therefore, the squeeze film damping force caused by the vibration of the cantilever with the sample affected the cantilever during the approach in tapping mode.

A Multi 75Al (Budget Sensors, Sofia, Bulgaria) cantilever was also used to test the squeeze film effect in the same environment. Its nominal elastic constant was 3 N/m, and its size was 225 μm × 28 μm × 3 μm. The quality factor was measured when the tip–sample distance was about 2 μm and when the sample was not placed under the cantilever. The corresponding quality factors at the same experimental conditions were 167.5 and 209.5, respectively. The results showed that the quality factor of the cantilever when the tip–sample distance was about 2 μm was much smaller than that of the cantilever when the sample was not placed under the cantilever.

**Figure 6:**
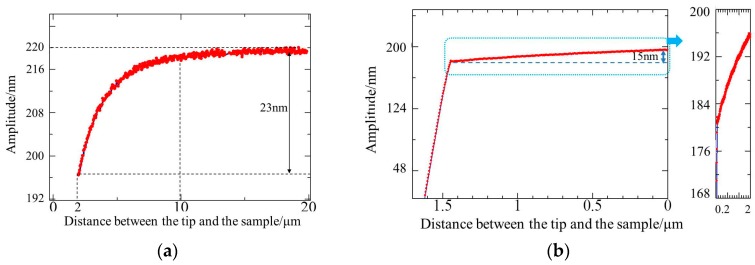
Approach curve of the 3 N/m cantilever in tapping mode (**a**) in the range of 2–20 μm (**b**) within 2 μm.

The approaching curve was tested in the range of 2–20 μm. The cantilever resonated with a frequency of 82.3 kHz and the free oscillation amplitude was about 220 nm. The deflection sensitivity was 13.1 V/μm. Experiment results are shown in Figure 6a. When the distance was wide, squeeze film damping did not exist. Therefore, the amplitude remained unchanged. As the cantilever approached the sample gradually, squeeze film damping was generated, which caused cantilever amplitude variation. As the cantilever approached further, the amplitude decreased significantly. The reduction was approximately 23 nm, which was about 10% of the free amplitude. Furthermore, the approach curve was tested when the tip-sample distance was within 2 μm, and the experimental results are shown in Figure 6b. When the tip–sample distance was smaller than 0.3 μm, the effect of Van der Waal’s force caused the amplitude to decrease rapidly. Prior to the effect of that force, the amplitude decreased by about 15 nm due to the effect of squeeze film damping in the range of 0.3–2 μm. From Figure 6a,b, the decreasing trend of the amplitude was consistent with the increase rule of the damping coefficient in Figure 2 when the tip–sample distance was within 0.3–10 μm. The total amplitude reduction was about 38 nm in the entire approaching process, which was about 15% of the free amplitude.

It can be seen that the amplitude variation trend in the experiments is consistent with the damping variation trend in theory. Experimental results also showed the influence of cantilever size on squeeze film damping. During the approach in the range of 2–20 μm, the reduction of larger cantilever caused by squeeze film damping was approximately 14% of the free amplitude, whereas the smaller cantilever was approximately 10% of the free amplitude.

The above phenomenon exists in the process of DPL. In DPL, modifications of the sample can be obtained by changing the modulation amplitude applied to the dither piezo that drives the cantilever oscillations. The signal used to write *V_w_* is several times larger than the signal used to read *V_r_*. Thus, when the cantilever is far from the surface, the writing free oscillation amplitude *A_wf_* is larger than the reading free oscillation amplitude *A_rf_* with the same factor. The efficiency of the writing process (i.e., the depth of the written structures) depends on the difference *A_w_* − *A_r_*, where *A_w_* and *A_r_* are the writing and reading amplitudes when the tip is close to the surface. This depends on *A_wf_* − *A_rf_* and the damping of the cantilever oscillations. Due to the squeeze film air damping of the cantilever and sample in the approach process, the actual writing and reading amplitudes *A_w_* and *A_r_* are different with the nominal *A_wf_* and *A_rf_*. For example, in the above experiment, the total amplitude reduction of the 3 N/m cantilever was about 15% of the free amplitude. Therefore, the *A_w_* is 85% of the *A_wf_* and the *A_r_* is 85% of the *A_rf_* if the cantilever is used in in DPL. Then, *A_w_* − *A_r_* = 0.85 × (*A_wf_* − *A_rf_*), which means that the actual depth *h_wa_* is 85% of the expected depth *h_we_*. The relative error can be calculated as follows:

*γ* = Δ*h_w_*/*h_e_* × 100% = (*h_wa_* − *h_we_*)/*h_we_* × 100% = 15% (11)

Obviously, the squeeze film damping effect causes the difference between the actual depth and expected depth of the written structure, and then the error is generated. The error is a system error and cannot be neglected for nanoscale patterning. Based on the analysis, the system error caused by the squeeze film damping effect can be corrected and the accuracy of the DPL can be improved.

## 4. Conclusions

Considering the features of the tapping cantilever in tapping mode AFM, the main factor of air squeeze film damping was studied, and the influence of damping on cantilever dynamics was analyzed. The cantilever–sample distance was small. Hence, cantilever vibration made air flow in and out of the gap between the cantilever and the sample, which led to squeeze film damping. Cantilever–sample distance is the main factor in damping. Experiments were performed using the tapping mode AFM. The amplitude variation trend in the experiments is consistent with the damping variation trend in theory. Theoretical analysis and experimental results showed that cantilever damping ratio increases and cantilever amplitude declines with the decreasing cantilever–sample distance. The amplitude reduction of the larger cantilever was more than that of the smaller cantilever. Squeeze film air damping exists in the approach and the indenting process. Therefore, squeeze film air damping influences the cantilever characteristic and the precision of the nanoscale patterning depth. This phenomenon also exists in the imaging process of tapping mode AFM and can reduce measurement precision. The error caused by air damping must be avoided or corrected in future work for accurate AFM patterning and measurement.
